# Evaluating adult forensic staff knowledge of olanzapine long-acting injection post injection syndrome: a service improvement project

**DOI:** 10.1192/bjo.2021.376

**Published:** 2021-06-18

**Authors:** Annalie Clark, Onagh Boyle, Suhanthini Farrell, Catrin Evans

**Affiliations:** Greater Manchester Mental Health NHS Foundation Trust

## Abstract

**Aims:**

Post injection syndrome (PIS) is a serious complication that can occur after Olanzapine Long Acting Injection (LAI). It can occur without any derangement in physical observations. It is important that patients are monitored appropriately following administration of Olanzapine LAI to ensure that symptoms of PIS are appropriately identified and managed. This project aimed to evaluate the current level of knowledge about PIS in two staff groups within an Adult Forensic Service – in-patient nursing staff and junior doctors and advanced practitioners (APs) providing medical cover to inpatient wards.

**Method:**

Electronic surveys evaluating knowledge about the symptoms of PIS, monitoring requirements and management of possible PIS were circulated to inpatient nursing staff, junior doctors and APs working within an Adult Forensic Service in the North West of England.

**Result:**

1) Nursing staff knowledge – 26 nursing staff completed the survey. 4.5% of nurses correctly identified all symptoms of PIS and 72.7% believed that tachycardia or hypotension occur in PIS. 22.7% of nurses identified the correct management plan if a patient feels unwell following Olanzapine LAI. 40.9% would only request a medical review if physical observations were abnormal. 2) Junior doctor and AP knowledge – 6 doctors and 6 advanced practitioners completed the survey. 17% of doctors and APs correctly identified all symptoms of PIS. 50% believed hypotension or tachycardia were symptoms of PIS. 25% of doctors and APs identified correct management of PIS and 16.7% believed that the patient should be managed on the psychiatric ward unless physical observations became abnormal.

**Conclusion:**

Levels of knowledge about the symptoms and management of PIS are low within this Adult Forensic Service. Knowledge of PIS and management of suspected PIS needs to be improved in nursing staff, junior doctors and advanced practitioners to ensure correct identification and safe management. In response to these findings, a care plan for monitoring of patients after Olanzapine LAI was developed. This included a structured monitoring proforma for completion post depot administration and instructions for managing suspected PIS. This care plan is kept in the front of the drug chart of all patients prescribed Olanzapine LAI. One-page educational summaries on PIS were written and circulated to nursing staff, junior doctors and APs. Information on Olanzapine LAI use and PIS were included in junior doctor induction materials and on-call handbook, to improve trainee awareness and knowledge.

